# Periodic Intermittent Adaptive Control with Saturation for Pinning Quasi-Consensus of Heterogeneous Multi-Agent Systems with External Disturbances

**DOI:** 10.3390/e25091266

**Published:** 2023-08-27

**Authors:** Bin Du, Quan Xu, Junfu Zhang, Yi Tang, Lei Wang, Ruihao Yuan, Yu Yuan, Jiaju An

**Affiliations:** School of Mechanical Engineering, Xihua University, Chengdu 610039, China; dubin97123456@163.com (B.D.);

**Keywords:** multi-agent systems, quasi-consensus, periodic intermittent, adaptive pinning control

## Abstract

A periodic intermittent adaptive control method with saturation is proposed to pin the quasi-consensus of nonlinear heterogeneous multi-agent systems with external disturbances in this paper. A new periodic intermittent adaptive control protocol with saturation is designed to control the internal coupling between the follower agents and the feedback gain between the leader and the follower. In particular, we use the saturation adaptive law: when the quasi-consensus error converges to a certain range, the adaptive coupling edge weight and the adaptive feedback gain will not be updated. Furthermore, we propose three saturated adaptive pinning control protocols. The quasi-consensus is achieved through its own pinning as long as the agents remain connected to each other. Using the Lyapunov function method and inequality technique, the convergence range of the quasi-consensus error of a heterogeneous multi-agent system is obtained. Finally, the rationality of the proposed control protocol is verified through numerical simulation. Theoretical derivation and simulation results show that the novel proposed periodic intermittent adaptive control method with saturation can successfully be used to achieve the pinning of quasi-consensus of nonlinear heterogeneous multi-agent systems.

## 1. Introduction

Scientists have conducted extensive research on the clustering phenomenon of various organisms in nature [1] and put forward the concept of multi-agent systems (MASs). Due to the high robustness of MASs’ distributed coordinated control, they have been widely used in practical engineering, including for UAV and robot formation, satellite orbit control, smart power grid, collaborative monitoring and other fields [2,3,4,5,6,7,8]; in addition, in-depth theoretical research has been conducted in control theory, physics, computer and other fields [9].

For MAS distributed coordination control, the consensus problem is the typical basis of multi-agent coordination control research. Methods of distributed consistent coordinated control of MASs have developed rapidly in recent years, for example, the consensus of transformation topology [10]; the consensus problem with communication delay [11]; the second-order, high-order and even fractional-order consensus [12]; and the consensus problem of finite time [13]. Multi-agent consensus control includes leaderless consensus and leader-following consensus. And the control protocol value of the latter is determined by the initial state of the agent. It has the advantage of a predetermined consensus value for control. Therefore, it has been studied extensively in recent years. In [14], the author uses Lyapunov stability theory to realize the leader-following consensus of second-order MASs. A unified framework of complex network synchronization and MAS consensus is established in [15]. In [16], output feedback and state feedback are used to study the consensus problem of leaders and followers. In [17], the sliding mode method is used to solve the bounded unknown input of leads. In [18], the author realizes the leader-following consensus for a single-integrator system.

However, the above research on MASs is based on the premise that all agents have the same dynamics. Of course, in practical engineering, it is unrealistic to require each agent to satisfy the same dynamics. Therefore, the research on heterogeneous multi-agent systems (HMASs) is more practical. The current complete consensus of HMASs is not well studied. In [19], the author parameterizes the unknown dynamic linear of agents to realize the consistent tracking of HMASs. In [20], the distributed consensus problem of HMASs with asymmetric input saturation is studied. However, in other studies, heterogeneity was transformed into homogeneity [21,22] or complex compensators were added to eliminate heterogeneity [23,24,25,26]. These methods cannot be applied in practical engineering because of their complexity. In fact, in practical engineering applications, only a certain error range is allowed. So, there is no need for complete consensus between HMASs. Therefore, the research on quasi-consensus (QC) of HMASs is of more practical significance. In [27,28], the definition of HMAS QC is proposed and expanded.

Subsequently, researchers began to achieve the QC of multi-agent systems through sampling data control, pulse control, adaptive control and other methods [29,30,31]. In particular, adaptive control is favored by researchers in the field of control because of its many advantages in realizing collaborative control, especially its low operating cost, fewer system requirements, high robustness and strong adaptability. Yu Wenwu et al. [29] further studied the problem of leader-following consensus of second-order multi-agents through the method of adaptive control. In [31], two new inequalities are proposed and an adaptive controller is designed to realize the QC of HMASs. However, the above adaptive control methods rely on continuous control, which is an obstacle to the application of control methods in practical engineering, and the proposed cost-saving adaptive control method will have great advantages. In addition, since periodic intermittent control is a discontinuous control method, continuous control is not required. So, the cost is greatly reduced, clearly being more in line with actual needs. Since intermittent control is activated only at work time, its fault tolerance is greatly increased, which is important for dealing with HMASs problems. Combining the adaptive method with the periodic intermittent control method could be an effective approach. Motivated by the application of adaptive control in integer and fractional complex dynamic networks [32,33,34,35], a control method based on periodic intermittent adaptive control is proposed to realize the QC of nonlinear HMASs with external disturbances in this paper. This control method realizes discontinuous control and provides more possibilities for realizing control cost savings.

## 2. Preliminary Preparation and Model Description

### 2.1. Graph Theory

A graph can be used to represent the topological relationship in an HMAS. An N-dimensional graph G={V,E,A} includes the nodes $V=(v_{1},v_{2},\ldots ,v_{N})$, which are connected to edges between different agents E⊆{(i,j)|i,j∈V,i≠j}E⊆{(i,j)|i,j∈V,i≠j} in the adjacency matrix $A={\left(a_{ij}\right)}_{N\times N}$. In a directed graph, the edge (i,j)∈E indicates that agent j can obtain information from agent i, but agent i cannot obtain information from agent j. For undirected graphs, the edge (i,j)∈E indicates that agent i and agent j can exchange information with each other. We assume that the topological connection is an undirected graph, and node i and node j are called neighbor nodes in this paper. The undirected connecting edge between node i and node j can be represented by $\left(v_{i},v_{j}\right)$ and $a_{ij}{=a}_{\mathrm{ji}}>0$ is the weight of undirected edges $\left(v_{i},v_{j}\right)$. When node i is not connected to node j, $a_{ij}{=a}_{\mathrm{ji}}=0$. The degree matrix $D=\mathrm{diag}(d_{i})$ with $d_{i}=\sum_{j\in N_{i}}a_{ij}$ and the Laplace matrix $L={\left(L_{ij}\right)}_{N\times N}$ with $L_{ij}=\sum_{j=1.j\neq i}^{N}a_{ij}$ and L=D-A.

**Lemma** **1** [36]. *The Laplacian matrix* L *of an undirected graph*  G *satisfies*

*1*. *The Laplacian matrix,* L*, is positive semi-definite, and its eigenvalues are* 0 *and positive.*

*2*. *The smallest nonzero eigenvalues *$\lambda_{2}\left(L\right)$ *satisfies*

$$\lambda_{2}\left(L\right){=}_{xT_{1}}\underset{N^{=0,x\neq 0}}{\min}\frac{x^{T}Lx}{x^{T}x}$$

*3*. *For any vector* $\eta ={\left(\eta_{1},\eta_{2},\ldots ,\eta_{n}\right)}^{T}\in R^{N}$*,*

$$\eta^{T}L\eta =\frac{1}{2}\sum_{i=1}^{N}\sum_{j=1}^{N}A_{ij}{\left(\eta_{i}-\eta_{j}\right)}^{2}$$

**Lemma** **2** [37]. *Let* *a continuous function* V:[μ,∞)→[0,∞)*, which satisfies*

(1) $$\dot{V}\left(t\right)\leq -g_{1}V\left(t\right)+\omega^{2}$$

If $g_{1}>0,\omega^{2}>0$, when t≥a:

(2) $$V\left(t\right)<V\left(a\right)\exp\{-g_{1}(t-a)\}+\frac{\omega^{2}}{g_{1}},t\geq a$$

**Lemma** **3** [38]. *For vector* $x,y\in R^{n}$*,* *there is a constant* γ>0 *, which makes the following inequality true:*

(3) $$2x^{T}y\leq \gamma x^{T}x+\gamma^{-1}y^{T}y$$

**Assumption** **1.** *Suppose the nonlinear function* f(t,⋅) *for vector* $\bar{\alpha },\bar{\beta }\in R^{n}$ *satisfies*

(4) $$\left||f\left(\bar{\alpha },t\right)-f\left(\bar{\beta },t\right)||\leq l||\bar{\alpha }-\bar{\beta }|\right|$$

where l is a positive constant.

**Assumption** **2.** *Suppose that the external disturbances is bounded and satisfies*

(5) $$\left|\right|\psi \left(t\right)-\varpi_{i}\left(t\right)\left|\right|\leq S_{i}$$

where $S_{i}>0$

**Assumption** **3.** *Suppose the network connection topology between the following multiple agents is undirected (* $L_{ij}=L_{ji}$ *)* *; each follower can obtain the state information of the agent with the coupling relationship and the leader agent at any time.*

**Definition** **1.** *For an HMAS, if each agent can satisfy the following inequality for any state variable of the system under the initial conditions, the entire HMAS is said to have reached QC:*

(6) $$\underset{t\to +\infty }{\lim}||z_{i}\left(t\right)-z_{0}\left(t\right)||\leq \delta ,i=1,2,\ldots ,N$$

### 2.2. Model Description

Consider an HMAS with 1+N multi-agents; the subscripts 0 and i are used to represent the leader and follower, respectively. The dynamic equations of the leader multi-agent and follower multi-agent are described as follows:

(7) $${\dot{z}}_{0}\left(t\right)=C_{0}z_{0}\left(t\right)+D_{0}f\left(z_{0}\left(t\right),t\right)+\psi \left(t\right)$$

(8) $${\dot{z}}_{i}\left(t\right)=C_{i}z_{i}\left(t\right)+D_{i}f\left(z_{i}\left(t\right),t\right)+\varpi_{i}\left(t\right)+u_{i}\left(t\right)$$

where $z_{i}\in R^{n}$ represents the follower state variable, $z_{0}\in R^{n}$ represents the leader state variable, $f:R^{n}\times R^{n}\times R^{+}\to R^{n}$ is a nonlinear function, $C_{0},D_{0}\in R^{n\times n}$ is the leader parameter matrix, $C_{i},D_{i}\in R^{n\times n}$ is the parameter matrix of the ith agent, $\varpi_{i}\left(t\right),\psi \left(t\right)\in R^{n}$ is the external time-varying disturbance, and $u_{i}\left(t\right)\in R^{n}$ is the control protocol.

The error is described as $\xi_{i}\left(t\right)=z_{i}\left(t\right)-z_{0}\left(t\right)$; as a result, the error model is as follows:

(9) $${\dot{\xi }}_{i}\left(t\right)=C_{i}\xi_{i}\left(t\right)+D_{i}f\left(\xi_{i}\left(t\right),t\right)+h_{i}\left(x_{0}\left(t\right),t\right)+\varpi_{i}\left(t\right)-\psi \left(t\right)-u_{i}\left(t\right)$$

where

$$f\left(\xi_{i}\left(t\right),t\right)=f\left(z_{i}\left(t\right),t\right)-f\left(z_{0}\left(t\right),t\right),\;h_{i}\left(z_{0}\left(t\right),t\right)=\left(C_{i}-C_{0}\right)z_{0}\left(t\right)+\left(D_{i}-D_{0}\right)f\left(z_{0}\left(t\right),t\right)$$

Consider the following control protocol for achieving QC between the leader state (7) and the follower state (8):

(10) $$u_{i}=\{\begin{array}{c} -c\sum_{j=1}^{N}L_{ij}(t)\left(z_{j}\left(t\right)-z_{i}\left(t\right)\right)-r_{i}(t)\left(z_{i}\left(t\right)-z_{0}\left(t\right)\right),nT\leq t\leq nT+\sigma T\\ 0,nT+\sigma T\leq t\leq \left(n+1\right)T \end{array}$$

where c represents the coupling strength of the communication topology in the HMASs.

For the control protocol (10), we designed the following adaptive law:

(11) $${\dot{r}}_{i}\left(t\right)=\gamma_{i}\pi_{i}e^{2\beta t}{\left(z_{i}\left(t\right)-z_{0}\left(t\right)\right)}^{T}\left(z_{i}\left(t\right)-z_{0}\left(t\right)\right)$$

(12) $${\dot{L}}_{ij}\left(t\right)=-\kappa_{ij}\pi_{i}e^{2\beta t}{\left(z_{i}\left(t\right)-z_{j}\left(t\right)\right)}^{T}\left(z_{i}\left(t\right)-z_{j}\left(t\right)\right),L_{ij}\left(0\right)=L_{ji}\left(0\right)>0,\left(i,j\right)\in E$$

(13) $$\pi_{i}=\{\begin{array}{c} 1,when||\xi ||>\varepsilon \\ 0,\begin{array}{cccc} & & & others \end{array} \end{array}$$

where $\gamma_{i,}\kappa_{ij}=\kappa_{ji}$ are positive constants.

**Remark** **1.** *The condition of convergence can be achieved quickly by using this adaptive law. But in this quasi-consensus study, the error converges to a certain range and does not become zero. Therefore, when the error reaches the allowable range, the adaptive law will continue to increase rapidly. This will increase the control cost in practical applications. Therefore, we designed an adaptive control protocol with saturation. When the error converges to our allowable range, the adaptive law becomes zero. In this case, the adaptive feedback gain and adaptive coupling side weight will not be updated, which greatly reduces the practical application cost. And when the error converges to the range that we allow, the error itself is small enough. Under the action of feedback gain and coupling side weight, the error can be controlled within the allowable range.*

In combination with (10)–(12), error model (9) is as follows:

(14) $${\dot{\xi }}_{i}=\{\begin{array}{c} C_{i}\xi_{i}\left(t\right)+D_{i}f\left(\xi_{i}\left(t\right),t\right)+h_{i}\left(z_{0}\left(t\right),t\right)+\varpi_{i}\left(t\right)-\psi \left(t\right)-c\sum_{j=1}^{N}L_{ij}\left(t\right)\xi_{j}\left(t\right)-r_{i}\left(t\right)\xi_{i}\left(t\right),nT\leq t\leq nT+\sigma T\\ C_{i}\xi_{i}\left(t\right)+D_{i}f\left(\xi_{i}\left(t\right),t\right)+h_{i}\left(z_{0}\left(t\right),t\right)+\varpi_{i}\left(t\right)-\psi \left(t\right),nT+\sigma T\leq t\leq \left(n+1\right)T \end{array}$$

## 3. Main Result

### 3.1. Adaptive Control Protocol

**Theorem** **1.** *If the HMAS satisfies Assumptions 1–4, the HMAS can achieve QC under the adaptive control protocol (10) and adaptive laws (11) and (12)*.

**Proof:**  When ||ξ||>ε, $\pi_{i}=1$, we construct the following Lyapunov function to achieve the QC of leader-following HMASs:

(15) $$V\left(t\right)=\frac{1}{2}\sum_{i=1}^{N}\xi_{i}^{T}\left(t\right)\xi_{i}\left(t\right)+\frac{1}{2}\sum_{i=1}^{N}\sum_{j=1}^{N}ce^{-2\beta t}\frac{{\left(L_{ij}\left(t\right)+L_{ij}\right)}^{2}}{2\kappa_{ij}}+\frac{1}{2}\sum_{i=1}^{N}ce^{-2\beta t}\frac{{\left(r_{i}\left(t\right)-r_{i}\right)}^{2}}{\gamma_{i}}$$

when nT≤t≤nT+σT.

Taking the derivative of (15), we can obtain

$$\begin{array}{l} \dot{V}\left(t\right)=\sum_{i=1}^{N}\xi_{i}^{T}\left(t\right){\dot{\xi }}_{i}\left(t\right)+\frac{1}{2}\sum_{i=1}^{N}\sum_{j=1}^{N}\left(-2\beta c\right)e^{-2\beta t}\frac{{\left(L_{ij}\left(t\right)+L_{ij}\right)}^{2}}{2\kappa_{ij}}+\sum_{i=1}^{N}ce^{-2\beta t}\frac{\left(L_{ij}\left(t\right)+L_{ij}\right)}{2\kappa_{ij}}\dot{L_{ij}}\left(t\right)\\ +\frac{1}{2}\sum_{i=1}^{N}\left(-2\beta c\right)e^{-2\beta t}\frac{{\left(r_{i}\left(t\right)-r_{i}\right)}^{2}}{\gamma_{i}}+\sum_{i=1}^{N}ce^{-2\beta t}\frac{\left(r_{i}\left(t\right)-r_{i}\right)}{\gamma_{i}}\dot{r_{i}}\left(t\right) \end{array}$$

$$\begin{array}{l} =\sum_{i=1}^{N}\xi_{i}^{T}\left(t\right)C_{i}\xi_{i}\left(t\right)+\sum_{i=1}^{N}\xi_{i}^{T}\left(t\right)D_{i}f\left(\xi_{i}\left(t\right),t\right)+\sum_{i=1}^{N}\xi_{i}^{T}\left(t\right)h_{i}\left(z_{0}\left(t\right),t\right)+\sum_{i=1}^{N}\xi_{i}^{T}\left(\varpi_{i}\left(t\right)-\psi \left(t\right)\right)\\ -\sum_{i=1}^{N}\xi_{i}^{T}\left(t\right)\left(c\sum_{j=1}^{N}L_{ij}\left(t\right)\xi_{j}\left(t\right)+r_{i}\left(t\right)\xi_{i}\left(t\right)\right)\\ +\frac{1}{2}\sum_{i=1}^{N}\sum_{j=1}^{N}\left(-2\beta c\right)e^{-2\beta t}\frac{{\left(L_{ij}\left(t\right)+L_{ij}\right)}^{2}}{2\kappa_{ij}}+\sum_{i=1}^{N}ce^{-2\beta t}\frac{\left(L_{ij}\left(t\right)+L_{ij}\right)}{2\kappa_{ij}}\left(-\kappa_{ij}e^{2\beta t}{\left(z_{i}\left(t\right)-z_{j}\left(t\right)\right)}^{T}\left(z_{i}\left(t\right)-z_{j}\left(t\right)\right)\right)\\ +\frac{1}{2}\sum_{i=1}^{N}\left(-2\beta c\right)e^{-2\beta t}\frac{{\left(r_{i}\left(t\right)-r_{i}\right)}^{2}}{\gamma_{i}}+\sum_{i=1}^{N}ce^{-2\beta t}\frac{\left(r_{i}\left(t\right)-r_{i}\right)}{\gamma_{i}}\left(\gamma_{i}e^{2\beta t}{\left(z_{i}\left(t\right)-z_{0}\left(t\right)\right)}^{T}\left(z_{i}\left(t\right)-z_{0}\left(t\right)\right)\right) \end{array}$$

(16) $$\begin{array}{l} =\sum_{i=1}^{N}\xi_{i}^{T}\left(t\right)C_{i}\xi_{i}\left(t\right)+\sum_{i=1}^{N}\xi_{i}^{T}\left(t\right)D_{i}f\left(\xi_{i}\left(t\right),t\right)+\sum_{i=1}^{N}\xi_{i}^{T}\left(t\right)h_{i}\left(z_{0}\left(t\right),t\right)+\sum_{i=1}^{N}\xi_{i}^{T}\left(\varpi_{i}\left(t\right)-\psi \left(t\right)\right)\\ -\sum_{i=1}^{N}\xi_{i}^{T}\left(t\right)\left(c\sum_{j=1}^{N}L_{ij}\left(t\right)\xi_{j}\left(t\right)+r_{i}\left(t\right)\xi_{i}\left(t\right)\right)\\ +\frac{1}{2}\sum_{i=1}^{N}\sum_{j=1}^{N}\left(-2\beta c\right)e^{-2\beta t}\frac{{\left(L_{ij}\left(t\right)+L_{ij}\right)}^{2}}{2\kappa_{ij}}-\frac{1}{2}\sum_{i=1}^{N}\sum_{j=1}^{N}c(L_{ij}\left(t\right)+L_{ij})\left({\left(z_{i}\left(t\right)-z_{j}\left(t\right)\right)}^{T}\left(z_{i}\left(t\right)-z_{j}\left(t\right)\right)\right)\\ +\frac{1}{2}\sum_{i=1}^{N}\left(-2\beta c\right)e^{-2\beta t}\frac{{\left(r_{i}\left(t\right)-r_{i}\right)}^{2}}{\gamma_{i}}+\sum_{i=1}^{N}c(r_{i}\left(t\right)-r_{i})\left({\left(z_{i}\left(t\right)-z_{0}\left(t\right)\right)}^{T}\left(z_{i}\left(t\right)-z_{0}\left(t\right)\right)\right) \end{array}$$

We can define the Laplacian matrix $\Omega ={\left(\tau_{ij}\right)}_{N\times N}$, where $\tau_{ij}=-L_{ij},i\neq j$ and

$\tau_{ii}=-\sum_{j=1,j\neq i}^{N}\tau_{ij}$, through Lemma 1, one can obtain

(17) $$\frac{1}{2}\sum_{i=1}^{N}\sum_{j=1}^{N}c(L_{ij}\left(t\right)+L_{ij})\left({\left(z_{i}\left(t\right)-z_{j}\left(t\right)\right)}^{T}\left(z_{i}\left(t\right)-z_{j}\left(t\right)\right)\right)=-c\sum_{i=1}^{N}\sum_{j=1}^{N}L_{ij}\left(t\right)\xi_{i}{}^{T}\xi_{j}+c\sum_{i=1}^{N}\sum_{j=1}^{N}\tau_{ij}\xi_{i}{}^{T}\xi_{j}$$

Then one can obtain

$$\begin{array}{l} \dot{V}\left(t\right)=\sum_{i=1}^{N}\xi_{i}^{T}\left(t\right)C_{i}\xi_{i}\left(t\right)+\sum_{i=1}^{N}\xi_{i}^{T}\left(t\right)D_{i}f\left(\xi_{i}\left(t\right),t\right)+\sum_{i=1}^{N}\xi_{i}^{T}\left(t\right)h_{i}\left(z_{0}\left(t\right),t\right)+\sum_{i=1}^{N}\xi_{i}^{T}\left(\varpi_{i}\left(t\right)-\psi \left(t\right)\right)\\ +\frac{1}{2}\sum_{i=1}^{N}\sum_{j=1}^{N}\left(-2\beta c\right)e^{-2\beta t}\frac{{\left(L_{ij}\left(t\right)+L_{ij}\right)}^{2}}{2\kappa_{ij}}-c\sum_{i=1}^{N}\sum_{j=1}^{N}\tau_{ij}\xi_{i}{}^{T}\xi_{j}\\ +\frac{1}{2}\sum_{i=1}^{N}\left(-2\beta c\right)e^{-2\beta t}\frac{{\left(r_{i}\left(t\right)-r_{i}\right)}^{2}}{\gamma_{i}}-c\sum_{i=1}^{N}r_{i}\xi_{i}{}^{T}\xi_{i} \end{array}$$

Through Lemma 3 and Assumption 1, one can obtain

(18) $$\begin{array}{l} \sum_{i=1}^{N}\xi_{i}^{T}\left(t\right)D_{i}f\left(\xi_{i}\left(t\right),t\right)=\sum_{i=1}^{N}e_{i}^{T}\left(t\right)D_{i}\left(f\left(z_{i}\left(t\right),t\right)-f\left(z_{0}\left(t\right),t\right)\right)\\ \leq \frac{1}{2}\sum_{i=1}^{N}\xi_{i}^{T}\left(t\right)D_{i}D_{i}{}^{T}\xi_{i}\left(t\right)+\frac{1}{2}\sum_{i=1}^{N}\left|\right|f\left(z_{i}\left(t\right),t\right)-f\left(z_{0}\left(t\right),t\right){\left|\right|}_{2}^{2}\\ \leq \frac{1}{2}\sum_{i=1}^{N}\xi_{i}^{T}\left(t\right)\left(D_{i}D_{i}{}^{T}+l^{2}I_{n}\right)\xi_{i}\left(t\right) \end{array}$$

and

(19) $$\sum_{i=1}^{N}\xi_{i}^{T}\left(t\right)h_{i}\left(z_{0}\left(t\right),t\right)\leq \frac{1}{2}\sum_{i=1}^{N}\xi_{i}^{T}\left(t\right)\xi_{i}\left(t\right)+\frac{1}{2}\sum_{i=1}^{N}\left|\right|h_{i}\left(z_{0}\left(t\right),t\right){\left|\right|}_{2}^{2}$$

and

(20) $$\sum_{i=1}^{N}\xi_{i}^{T}\left(\varpi_{i}\left(t\right)-\psi \left(t\right)\right)\leq \frac{1}{2}\sum_{i=1}^{N}\xi_{i}^{T}\xi_{i}+\frac{1}{2}\sum_{i=1}^{N}S^{2}{}_{i}$$

then

$$\begin{array}{l} \dot{V}\left(t\right)\leq \sum_{i=1}^{N}\xi_{i}^{T}\left(t\right)C_{i}\xi_{i}\left(t\right)+\frac{1}{2}\sum_{i=1}^{N}\xi_{i}^{T}\left(t\right)\left(D_{i}D_{i}{}^{T}+\left(l^{2}+2\right)I_{n}-2r_{i}I_{n}\right)\xi_{i}\left(t\right)\\ +\frac{1}{2}\sum_{i=1}^{N}\left|\right|h_{i}\left(z_{0}\left(t\right),t\right){\left|\right|}_{2}^{2}+\frac{1}{2}\sum_{i=1}^{N}S^{2}{}_{i}-c\sum_{i=1}^{N}\sum_{j=1}^{N}\tau_{ij}\xi_{i}^{T}\left(t\right)\xi_{j}\left(t\right)\\ +\frac{1}{2}\sum_{i=1}^{N}\sum_{j=1}^{N}\left(-2\beta c\right)e^{-2\beta t}\frac{{\left(L_{ij}\left(t\right)+L_{ij}\right)}^{2}}{2\kappa_{ij}}+\frac{1}{2}\sum_{i=1}^{N}\left(-2\beta c\right)e^{-2\beta t}\frac{{\left(r_{i}\left(t\right)-r_{i}\right)}^{2}}{\gamma_{i}} \end{array}$$

We define $\frac{1}{2}\left|\right|h\left(z_{0}\left(t\right),t\right){\left|\right|}_{2}^{2}+\frac{1}{2}\sum_{i=1}^{N}S^{2}{}_{i}=\omega^{2}$; let Λ be the diagonal matrix of Ω. There is a unitary matrix $U=\left(u_{1},\ldots ,u_{N}\right)$, so that $U^{T}\Omega U=\Lambda$. Let $y\left(t\right)=\left(U^{T}⊗I_{n}\right)\xi \left(t\right)$, so that

(21) $$\begin{array}{l} \dot{V}\left(t\right)\leq \xi^{T}\left(t\right)\left(C+\frac{1}{2}\left(DD^{T}+I_{N}⊗\left(l^{2}+2\right)I_{n}-2\left(R⊗I_{n}\right)\right)-c\left(\Omega ⊗I_{n}\right)\right)\xi \left(t\right)+\omega^{2}\\ +\frac{1}{2}\sum_{i=1}^{N}\sum_{j=1}^{N}\left(-2\beta c\right)e^{-2\beta t}\frac{{\left(L_{ij}\left(t\right)+L_{ij}\right)}^{2}}{2\kappa_{ij}}+\frac{1}{2}\sum_{i=1}^{N}\left(-2\beta c\right)e^{-2\beta t}\frac{{\left(r_{i}\left(t\right)-r_{i}\right)}^{2}}{\gamma_{i}}\\ =\xi^{T}\left(t\right)\left(C+\frac{1}{2}\left(DD^{T}+I_{N}⊗\left(l^{2}+2\right)I_{n}-2\left(R⊗I_{n}\right)\right)\right)\xi \left(t\right)-cy^{T}\left(t\right)\left(\Lambda ⊗I_{n}\right)y\left(t\right)+\omega^{2}\\ +\frac{1}{2}\sum_{i=1}^{N}\sum_{j=1}^{N}\left(-2\beta c\right)e^{-2\beta t}\frac{{\left(L_{ij}\left(t\right)+L_{ij}\right)}^{2}}{2\kappa_{ij}}+\frac{1}{2}\sum_{i=1}^{N}\left(-2\beta c\right)e^{-2\beta t}\frac{{\left(r_{i}\left(t\right)-r_{i}\right)}^{2}}{\gamma_{i}} \end{array}$$

where $C=diag\left(C_{1},C_{2},\ldots ,C_{N}\right)$, $D=diag\left(D_{1},D_{2},\ldots ,D_{N}\right)$, $R=diag\left(r_{1},r_{2},\ldots ,r_{N}\right)$.

Through Lemma 1, since $I_{n}$ is positive definite, we can obtain $y^{T}\left(t\right)\left(\Lambda ⊗I_{n}\right)y\left(t\right)\geq \lambda_{2}\left(\Omega \right)y^{T}\left(t\right)\left(I_{N}⊗I_{n}\right)y\left(t\right)$; hence,

$$\begin{array}{l} \dot{V}\left(t\right)\leq \xi^{T}\left(t\right)\left(C+\frac{1}{2}\left(DD^{T}+I_{N}⊗\left(l^{2}+2\right)I_{n}-2\left(R⊗I_{n}\right)\right)\right)\xi \left(t\right)-\lambda_{2}\left(\Omega \right)y^{T}\left(t\right)\left(I_{N}⊗I_{n}\right)y\left(t\right)+\omega^{2}\\ +\frac{1}{2}\sum_{i=1}^{N}\sum_{j=1}^{N}\left(-2\beta c\right)e^{-2\beta t}\frac{{\left(L_{ij}\left(t\right)+L_{ij}\right)}^{2}}{2\kappa_{ij}}+\frac{1}{2}\sum_{i=1}^{N}\left(-2\beta c\right)e^{-2\beta t}\frac{{\left(r_{i}\left(t\right)-r_{i}\right)}^{2}}{\gamma_{i}}\\ =\xi^{T}\left(t\right)\left(C+\frac{1}{2}\left(DD^{T}+I_{N}⊗\left(l^{2}+2\right)I_{n}-2\left(R⊗I_{n}\right)\right)\right)\xi \left(t\right)\\ -c\lambda_{2}\left(\Omega \right)\xi^{T}\left(t\right)\left(U⊗I_{n}\right)\left(I_{N}⊗I_{n}\right)\left(U^{T}⊗I_{n}\right)\xi \left(t\right)+\omega^{2}\\ +\frac{1}{2}\sum_{i=1}^{N}\sum_{j=1}^{N}\left(-2\beta c\right)e^{-2\beta t}\frac{{\left(L_{ij}\left(t\right)+L_{ij}\right)}^{2}}{2\kappa_{ij}}+\frac{1}{2}\sum_{i=1}^{N}\left(-2\beta c\right)e^{-2\beta t}\frac{{\left(r_{i}\left(t\right)-r_{i}\right)}^{2}}{\gamma_{i}}\\ =\xi^{T}\left(t\right)\left(C+\frac{1}{2}\left(DD^{T}+I_{N}⊗\left(l^{2}+2\right)I_{n}-2\left(R⊗I_{n}\right)\right)-c\lambda_{2}\left(\Omega \right)\left(I_{N}⊗I_{n}\right)\right)\xi \left(t\right)+\omega^{2}\\ +\frac{1}{2}\sum_{i=1}^{N}\sum_{j=1}^{N}\left(-2\beta c\right)e^{-2\beta t}\frac{{\left(L_{ij}\left(t\right)+L_{ij}\right)}^{2}}{2\kappa_{ij}}+\frac{1}{2}\sum_{i=1}^{N}\left(-2\beta c\right)e^{-2\beta t}\frac{{\left(r_{i}\left(t\right)-r_{i}\right)}^{2}}{\gamma_{i}} \end{array}$$

$$\begin{array}{l} \dot{V}\left(t\right)\leq \xi^{T}\left(t\right)\left(C+\frac{1}{2}\left(DD^{T}+I_{N}⊗\left(l^{2}+2\right)I_{n}-2\left(R⊗I_{n}\right)\right)-c\lambda_{2}\left(\Omega \right)\left(I_{N}⊗I_{n}\right)+\beta \left(I_{N}⊗I_{n}\right)\right)\xi \left(t\right)\\ -\beta \xi^{T}\left(t\right)\xi \left(t\right)+\omega^{2}+\frac{1}{2}\sum_{i=1}^{N}\sum_{j=1}^{N}\left(-2\beta c\right)e^{-2\beta t}\frac{{\left(L_{ij}\left(t\right)+L_{ij}\right)}^{2}}{2\kappa_{ij}}+\frac{1}{2}\sum_{i=1}^{N}\left(-2\beta c\right)e^{-2\beta t}\frac{{\left(r_{i}\left(t\right)-r_{i}\right)}^{2}}{\gamma_{i}} \end{array}$$

We can choose $L_{ij}$ and $r_{i}$, which are large enough to meet the conditions:

$C+\frac{1}{2}\left(DD^{T}+I_{N}⊗\left(l^{2}+2\right)I_{n}-2\left(R⊗I_{n}\right)\right)$$-c\lambda_{2}\left(\Omega \right)\left(I_{N}⊗I_{n}\right)+\beta \left(I_{N}⊗I_{n}\right)\leq 0$. One can obtain

$$\begin{array}{l} \dot{V}\left(t\right)\leq -\beta \sum_{i=1}^{N}\xi_{i}{}^{T}\left(t\right)\xi_{i}\left(t\right)+\frac{1}{2}\sum_{i=1}^{N}\sum_{j=1}^{N}\left(-2\beta c\right)e^{-2\beta t}\frac{{\left(L_{ij}\left(t\right)+L_{ij}\right)}^{2}}{2\kappa_{ij}}+\frac{1}{2}\sum_{i=1}^{N}\left(-2\beta c\right)e^{-2\beta t}\frac{{\left(r_{i}\left(t\right)-r_{i}\right)}^{2}}{\gamma_{i}}\\ +\omega^{2}\\ =-\beta \sum_{i=1}^{N}\xi_{i}{}^{T}\left(t\right)\xi_{i}\left(t\right)-\sum_{i=1}^{N}\sum_{j=1}^{N}\left(\beta c\right)e^{-2\beta t}\frac{{\left(L_{ij}\left(t\right)+L_{ij}\right)}^{2}}{2\kappa_{ij}}-\sum_{i=1}^{N}\left(\beta c\right)e^{-2\beta t}\frac{{\left(r_{i}\left(t\right)-r_{i}\right)}^{2}}{\gamma_{i}}+\omega^{2}\\ =-2\beta (\frac{1}{2}\sum_{i=1}^{N}\xi_{i}{}^{T}\left(t\right)\xi_{i}\left(t\right)+\frac{1}{2}\sum_{i=1}^{N}\sum_{j=1}^{N}ce^{-2\beta t}\frac{{\left(L_{ij}\left(t\right)+L_{ij}\right)}^{2}}{2\kappa_{ij}}+\frac{1}{2}\sum_{i=1}^{N}ce^{-2\beta t}\frac{{\left(r_{i}\left(t\right)-r_{i}\right)}^{2}}{\gamma_{i}})+\omega^{2} \end{array}$$

(22) $$=-g_{1}V\left(t\right)+\omega^{2}$$

where $g_{1}=2\beta$.

when nT+σT≤t≤(n+1)T.

Similar to the discussion above, we have

$$\begin{array}{l} \dot{V}\left(t\right)\leq \xi^{T}\left(t\right)\left(C+\frac{1}{2}\left(DD^{T}+I_{N}⊗\left(l^{2}+2\right)I_{n}\right)\right)\xi \left(t\right)+\omega^{2}\\ +\frac{1}{2}\sum_{i=1}^{N}\sum_{j=1}^{N}\left(-2\beta c\right)e^{-2\beta t}\frac{{\left(L_{ij}\left(t\right)+L_{ij}\right)}^{2}}{2\kappa_{ij}}+\frac{1}{2}\sum_{i=1}^{N}\left(-2\beta c\right)e^{-2\beta t}\frac{{\left(r_{i}\left(t\right)-r_{i}\right)}^{2}}{\gamma_{i}} \end{array}$$

(23) $$\begin{array}{l} \dot{V}\left(t\right)\leq \xi^{T}\left(t\right)\left(C+\frac{1}{2}\left(DD^{T}+I_{N}⊗\left(l^{2}+2\right)I_{n}\right)+\left(\beta -d\right)\left(I_{N}⊗I_{n}\right)\right)\xi \left(t\right)\\ +\left(d-\beta \right)\xi^{T}\left(t\right)\xi \left(t\right)+\omega^{2}+\frac{1}{2}\sum_{i=1}^{N}\sum_{j=1}^{N}\left(-2\beta c\right)e^{-2\beta t}\frac{{\left(L_{ij}\left(t\right)+L_{ij}\right)}^{2}}{2\kappa_{ij}}\\ +\frac{1}{2}\sum_{i=1}^{N}\left(-2\beta c\right)e^{-2\beta t}\frac{{\left(r_{i}\left(t\right)-r_{i}\right)}^{2}}{\gamma_{i}} \end{array}$$

where d−β>0. One can obtain

$$\begin{array}{l} \dot{V}\left(t\right)\leq \xi^{T}\left(t\right)\left(C+\frac{1}{2}\left(DD^{T}+I_{N}⊗\left(l^{2}+2\right)I_{n}\right)+\left(\beta -d\right)\left(I_{N}⊗I_{n}\right)\right)\xi \left(t\right)+\left(d-\beta \right)\xi^{T}\left(t\right)\xi \left(t\right)+\omega^{2}\\ +\sum_{i=1}^{N}\sum_{j=1}^{N}\left(-\beta c\right)e^{-2\beta t}\frac{{\left(L_{ij}\left(t\right)+L_{ij}\right)}^{2}}{2\kappa_{ij}}+\sum_{i=1}^{N}\left(-\beta c\right)e^{-2\beta t}\frac{{\left(r_{i}\left(t\right)-r_{i}\right)}^{2}}{\gamma_{i}}\\ +\sum_{i=1}^{N}\sum_{j=1}^{N}dce^{-2\beta t}\frac{{\left(L_{ij}\left(t\right)+L_{ij}\right)}^{2}}{2\kappa_{ij}}+\sum_{i=1}^{N}dce^{-2\beta t}\frac{{\left(r_{i}\left(t\right)-r_{i}\right)}^{2}}{\gamma_{i}} \end{array}$$

We choose d,β to meet the conditions . One can obtain

$$\begin{array}{l} \dot{V}\left(t\right)\leq \xi^{T}\left(t\right)\left(C+\frac{1}{2}\left(DD^{T}+I_{N}⊗\left(l^{2}+2\right)I_{n}\right)+\left(\beta -d\right)\left(I_{N}⊗I_{n}\right)\right)\xi \left(t\right)\\ +\left(d-\beta \right)\xi^{T}\left(t\right)\xi \left(t\right)+\omega^{2}+\sum_{i=1}^{N}\sum_{j=1}^{N}\left(d-\beta \right)ce^{-2\beta t}\frac{{\left(L_{ij}\left(t\right)+L_{ij}\right)}^{2}}{2\kappa_{ij}}\\ +\sum_{i=1}^{N}\left(d-\beta \right)ce^{-2\beta t}\frac{{\left(r_{i}\left(t\right)-r_{i}\right)}^{2}}{\gamma_{i}} \end{array}$$

(24) $$\begin{array}{l} \leq \left(d-\beta \right)\sum_{i=1}^{N}\xi_{i}^{T}\left(t\right)\xi_{i}\left(t\right)+\sum_{i=1}^{N}\sum_{j=1}^{N}\left(d-\beta \right)ce^{-2\beta t}\frac{{\left(L_{ij}\left(t\right)+L_{ij}\right)}^{2}}{2\kappa_{ij}}\\ +\sum_{i=1}^{N}\left(d-\beta \right)ce^{-2\beta t}\frac{{\left(r_{i}\left(t\right)-r_{i}\right)}^{2}}{\gamma_{i}}+\omega^{2}\\ =2\left(d-\beta \right)\left(\frac{1}{2}\sum_{i=1}^{N}\xi_{i}^{T}\left(t\right)\xi_{i}\left(t\right)+\frac{1}{2}\sum_{i=1}^{N}\sum_{j=1}^{N}ce^{-2\beta t}\frac{{\left(L_{ij}\left(t\right)+L_{ij}\right)}^{2}}{2\kappa_{ij}}+\frac{1}{2}\sum_{i=1}^{N}ce^{-2\beta t}\frac{{\left(r_{i}\left(t\right)-r_{i}\right)}^{2}}{\gamma_{i}}\right)\\ +\omega^{2}\\ {=g}_{2}V\left(t\right)+\omega^{2} \end{array}$$

where $g_{2}=2\left(d-\beta \right)$.

Combining (22) and (24), we have

(25) $$\{\begin{array}{c} \dot{V}\left(t\right)\leq -g_{1}V\left(t\right)+\omega^{2},nT\leq t\leq nT+\sigma T\\ \dot{V}\left(t\right)\leq g_{2}V\left(t\right)+\omega^{2},nT+\sigma T\leq t\leq \left(n+1\right)T \end{array}$$

through Lemma 2, when nT≤t≤nT+σT, we have the following:

(26) $$V\left(\xi \left(t\right)\right)\leq V\left(\xi \left(nT\right)\right)\exp\left(-g_{1}\left(t-nT\right)\right)+\frac{\lambda_{1}^{-1}\omega^{2}}{g_{1}}$$

Simultaneously, when nT+σT≤t≤(n+1)T, we have

(27) $$V\left(\xi \left(t\right)\right)\leq V\left(\xi \left(nT+\sigma T\right)\right)\exp\left(g_{2}\left(t-nT-\sigma T\right)\right)-\frac{\lambda_{1}^{-1}\omega^{2}}{g_{2}}$$

Combining this with (27), we have

(28) $$\{\begin{array}{c} V\left(t\right)\leq V\left(nT\right)\exp\left(-g_{1}\left(t-nT\right)\right)+\frac{\omega^{2}}{g_{1}},nT\leq t\leq nT+\sigma T\\ V\left(t\right)\leq V\left(nT+\sigma T\right)\exp\left(g_{2}\left(t-nT-\sigma T\right)\right)-\frac{\omega^{2}}{g_{2}},nT+\sigma T\leq t\leq \left(n+1\right)T \end{array}$$

Similar to the discussion in [37], when t≥0 if $1>g_{1}\sigma -g_{2}\left(1-\sigma \right)>0$, we obtain the following inequality:

(29) $$V\left(t\right)\leq V\left(0\right)\exp\left(-g_{1}\sigma t+g_{2}\left(1-\sigma \right)t\right)+\frac{\omega^{2}}{g_{1}}\left(1+\sum_{i=1}^{n}\exp\left(ig_{2}\left(T-\sigma T\right)-ig_{1}\sigma T\right)\right)$$

So, we can obtain:

$$\begin{array}{l} \frac{1}{2}\sum_{i=1}^{N}\xi_{i}^{T}\left(t\right)\xi_{i}\left(t\right)+\frac{1}{2}\sum_{i=1}^{N}\sum_{j=1}^{N}ce^{-2\beta t}\frac{{\left(L_{ij}\left(t\right)+L_{ij}\right)}^{2}}{2\eta_{i}}+\frac{1}{2}\sum_{i=1}^{N}ce^{-2\beta t}\frac{{\left(r_{i}\left(t\right)-r_{i}\right)}^{2}}{\gamma_{i}}\\ \leq V\left(0\right)\exp\left(-g_{1}\sigma t+g_{2}\left(1-\sigma \right)t\right)+\frac{\omega^{2}}{g_{1}}\left(1+\sum_{i=1}^{n}\exp\left(ig_{2}\left(T-\sigma T\right)-ig_{1}\sigma T\right)\right) \end{array}$$

(30) $$\frac{1}{2}\left|\right|\xi |{|}_{2}{}^{2}\leq V\left(0\right)\exp\left(-g_{1}\sigma t+g_{2}\left(1-\sigma \right)t\right)+\frac{\omega^{2}}{g_{1}}\left(1+\sum_{i=1}^{n}\exp\left(ig_{2}\left(T-\sigma T\right)-ig_{1}\sigma T\right)\right)$$

When N→+∞, one has:

(31) $$1+\sum_{i=1}^{n}\exp\left(ig_{2}\left(T-\sigma T\right)-ig_{1}\sigma T\right)\to \frac{1}{1-\exp\left(g_{2}\left(T-\sigma T\right)-g_{1}\sigma T\right)}$$

Then, the margin of error convergence can be obtained:

(32) $$\left\|\xi \left(t\right)\right\|\leq \sqrt{\frac{2\omega^{2}}{g_{1}}\left(\frac{1}{1-\exp\left(g_{2}\left(T-\sigma T\right)-g_{1}\sigma T\right)}\right)}$$

So far, it has been proved that the leader (7) and follower system (8) achieve QC, and the error range bounds of consensus are obtained. □

### 3.2. Adaptive Pinning Control

The control protocol (10) and adaptive laws (11) and (12) are used to control the whole situation. Each follower exchanges information with the leader. However, it is impractical and costly in practical engineering applications. In practical engineering, under large-scale tracking control, it is exceedingly expensive to maintain the information exchange between the leader and all followers, which will greatly hinder the application of distributed control methods. Therefore, this paper will continue to study the pinning control protocol. We only use adaptive laws for partial coupling topologies and the leader only interacts with some followers. We propose three pinning control schemes.

**Pinning** **1.** *We use the following control protocol and adaptive laws (11) and (12).*

(33) $$u_{i}=\{\begin{array}{c} -c\sum_{j=1}^{N}L_{ij}(t)\left(z_{j}\left(t\right)-z_{i}\left(t\right)\right)-\partial_{i}r_{i}(t)\left(z_{i}\left(t\right)-z_{0}\left(t\right)\right),nT\leq t\leq nT+\sigma T\\ 0,nT+\sigma T\leq t\leq \left(n+1\right)T \end{array}$$

where $\partial_{i}=1$ for $i=1,2,\ldots ,N_{S}$ and $\partial_{i}=0$ for $i=N_{S}+1,\ldots ,N$.

**Theorem** **2.** *If the HMAS satisfies Assumptions 1–4, the HMAS can achieve QC under the adaptive control protocol (33) and adaptive laws (11) and (12)*.

**Proof.**  When ||ξ||>ε, $\pi_{i}=1$. We construct the following Lyapunov function to achieve the QC of leader-following HMASs:

(34) $$V\left(t\right)=\frac{1}{2}\sum_{i=1}^{N}\xi_{i}^{T}\left(t\right)\xi_{i}\left(t\right)+\frac{1}{2}\sum_{i=1}^{N}\sum_{j=1}^{N}ce^{-2\beta t}\frac{{\left(L_{ij}\left(t\right)+L_{ij}\right)}^{2}}{2\kappa_{ij}}+\frac{1}{2}\sum_{i=1}^{N_{S}}ce^{-2\beta t}\frac{{\left(r_{i}\left(t\right)-r_{i}\right)}^{2}}{\gamma_{i}}$$

when nT≤t≤nT+σT.

Taking the derivative of (34), we have

$$\begin{array}{l} \dot{V}\left(t\right)=\sum_{i=1}^{N}\xi_{i}^{T}\left(t\right){\dot{\xi }}_{i}\left(t\right)+\frac{1}{2}\sum_{i=1}^{N}\sum_{j=1}^{N}\left(-2\beta c\right)e^{-2\beta t}\frac{{\left(L_{ij}\left(t\right)+L_{ij}\right)}^{2}}{2\kappa_{ij}}+\sum_{i=1}^{N}ce^{-2\beta t}\frac{\left(L_{ij}\left(t\right)+L_{ij}\right)}{2\kappa_{ij}}\dot{L_{ij}}\left(t\right)\\ +\frac{1}{2}\sum_{i=1}^{N_{S}}\left(-2\beta c\right)e^{-2\beta t}\frac{{\left(r_{i}\left(t\right)-r_{i}\right)}^{2}}{\gamma_{i}}+\sum_{i=1}^{N_{S}}ce^{-2\beta t}\frac{\left(r_{i}\left(t\right)-r_{i}\right)}{\gamma_{i}}\dot{r_{i}}\left(t\right) \end{array}$$

$$\begin{array}{l} \dot{V}\left(t\right)\leq \sum_{i=1}^{N}\xi_{i}^{T}\left(t\right)C_{i}\xi_{i}\left(t\right)+\frac{1}{2}\sum_{i=1}^{N}\xi_{i}^{T}\left(t\right)\left(D_{i}D_{i}{}^{T}+\left(l^{2}+2\right)I_{n}-2\partial_{i}r_{i}I_{n}\right)\xi_{i}\left(t\right)-c\sum_{i=1}^{N}\sum_{j=1}^{N}\tau_{ij}\xi_{i}^{T}\left(t\right)\xi_{j}\left(t\right)+\omega^{2}\\ +\frac{1}{2}\sum_{i=1}^{N}\sum_{j=1}^{N}\left(-2\beta c\right)e^{-2\beta t}\frac{{\left(L_{ij}\left(t\right)+L_{ij}\right)}^{2}}{2\kappa_{ij}}+\frac{1}{2}\sum_{i=1}^{N_{S}}\left(-2\beta c\right)e^{-2\beta t}\frac{{\left(r_{i}\left(t\right)-r_{i}\right)}^{2}}{\gamma_{i}} \end{array}$$

$$\begin{array}{l} \dot{V}\left(t\right)\leq \xi^{T}\left(t\right)\left(C+\frac{1}{2}\left(DD^{T}+I_{N}⊗\left(l^{2}+2\right)I_{n}-2\left(\tilde{R}⊗I_{n}\right)\right)\right)\xi \left(t\right)\\ -\xi^{T}\left(t\right)\left(c\lambda_{2}\left(\Omega \right)\left(I_{N}⊗I_{n}\right)+\beta \left(I_{N}⊗I_{n}\right)\right)\xi \left(t\right)-\beta \xi^{T}\left(t\right)\xi \left(t\right)+\omega^{2}\\ +\frac{1}{2}\sum_{i=1}^{N}\sum_{j=1}^{N}\left(-2\beta c\right)e^{-2\beta t}\frac{{\left(L_{ij}\left(t\right)+L_{ij}\right)}^{2}}{2\kappa_{ij}}+\frac{1}{2}\sum_{i=1}^{N_{S}}\left(-2\beta c\right)e^{-2\beta t}\frac{{\left(r_{i}\left(t\right)-r_{i}\right)}^{2}}{\gamma_{i}}\\ \leq -2\beta \left(\frac{1}{2}\sum_{i=1}^{N}\xi_{i}^{T}\left(t\right)\xi_{i}\left(t\right)+\frac{1}{2}\sum_{i=1}^{N}\sum_{j=1}^{N}ce^{-2\beta t}\frac{{\left(L_{ij}\left(t\right)+L_{ij}\right)}^{2}}{2\kappa_{ij}}+\frac{1}{2}\sum_{i=1}^{N_{S}}ce^{-2\beta t}\frac{{\left(r_{i}\left(t\right)-r_{i}\right)}^{2}}{\gamma_{i}}\right)+\omega^{2}\\ =-g_{1}V\left(t\right)+\omega^{2} \end{array}$$

where $\tilde{R}=diag\left(r_{1},r_{2},\ldots ,r_{N_{S}},0,\ldots ,0\right)$.

When nT+σT≤t≤(n+1)T, it is similar to Theorem 1. Hence, we have

(35) $$\{\begin{array}{c} \dot{V}\left(t\right)\leq -g_{1}V\left(t\right)+\omega^{2},nT\leq t\leq nT+\sigma T\\ \dot{V}\left(t\right)\leq g_{2}V\left(t\right)+\omega^{2},nT+\sigma T\leq t\leq \left(n+1\right)T \end{array}$$

The rest of the proof is the same as Theorem 1. □

**Pinning** **2.** *We use the control protocol (10) and adaptive law (11) and the following adaptive laws:*

(36) $${\dot{L}}_{ij}\left(t\right)=-\kappa_{ij}e^{2\beta t}{\left(z_{i}\left(t\right)-z_{j}\left(t\right)\right)}^{T}\left(z_{i}\left(t\right)-z_{j}\left(t\right)\right),L_{ij}\left(0\right)=L_{ji}\left(0\right)>0,\left(i,j\right)\in \tilde{E}$$

(37) $$\pi_{i}=\{\begin{array}{c} 1,when||\xi ||>\varepsilon \\ 0,\begin{array}{cccc} & & & others \end{array} \end{array}$$

where $\tilde{E}$ is the subset of E, and $\tilde{E}$ is connected.

**Theorem** **3.** *If the* *HMAS satisfies Assumptions 1–4, the HMAS can achieve QC under the adaptive control protocol (10)* *and adaptive laws (11* *) and (36).*

**Proof.**  When ||ξ||>ε, $\pi_{i}=1$. We construct the following Lyapunov function to achieve the QC of leader-following HMASs:

(38) $$V\left(t\right)=\frac{1}{2}\sum_{i=1}^{N}\xi_{i}^{T}\left(t\right)\xi_{i}\left(t\right)+\frac{1}{2}\sum_{i=1}^{N}\sum_{\left(i,j\right)\in \tilde{E}}^{}ce^{-2\beta t}\frac{{\left(L_{ij}\left(t\right)+\tilde{L_{ij}}\right)}^{2}}{2\kappa_{ij}}+\frac{1}{2}\sum_{i=1}^{N}ce^{-2\beta t}\frac{{\left(r_{i}\left(t\right)-r_{i}\right)}^{2}}{\gamma_{i}}$$

where $\tilde{L_{ij}}=\tilde{L_{ji}}>0,\left(i,j\right)\in \tilde{E}$ and $L_{ij}=0,\left(i\neq j\right)$. Let $\tilde{\Omega }={\left(\tilde{\tau_{ij}}\right)}_{N\times N}$, where $\tilde{\tau_{ij}}=\tilde{L_{ij}}.i\neq j$ and $\tilde{\tau_{ii}}=-\sum_{j=1,j\neq i}^{N}\tilde{\tau_{ij}}$; then,

(39) $$G_{ij}=\{\begin{array}{c} L_{ij}\left(0\right),\left(i,j\right)\in E-\tilde{E}\\ -\sum_{j=1,j\neq i}^{N}L_{ij}\left(0\right),i=j\\ 0,other \end{array}$$

when nT≤t≤nT+σT.

Taking the derivative of (39), we have

$$\begin{array}{l} \dot{V}\left(t\right)=\sum_{i=1}^{N}\xi_{i}^{T}\left(t\right){\dot{\xi }}_{i}\left(t\right)+\frac{1}{2}\sum_{i=1}^{N}\sum_{\left(i,j\right)\in \tilde{E}}^{}\left(-2\beta c\right)e^{-2\beta t}\frac{{\left(L_{ij}\left(t\right)+\tilde{L_{ij}}\right)}^{2}}{2\kappa_{ij}}+\sum_{i=1}^{N}\sum_{\left(i,j\right)\in \tilde{E}}^{}ce^{-2\beta t}\frac{\left(L_{ij}\left(t\right)+\tilde{L_{ij}}\right)}{2\kappa_{ij}}\dot{L_{ij}}\left(t\right)\\ +\frac{1}{2}\sum_{i=1}^{N}\left(-2\beta c\right)e^{-2\beta t}\frac{{\left(r_{i}\left(t\right)-r_{i}\right)}^{2}}{\gamma_{i}}+\sum_{i=1}^{N}ce^{-2\beta t}\frac{\left(r_{i}\left(t\right)-r_{i}\right)}{\gamma_{i}}\dot{r_{i}}\left(t\right) \end{array}$$

$$\begin{array}{l} \dot{V}\left(t\right)\leq \sum_{i=1}^{N}\xi_{i}^{T}\left(t\right)C_{i}\xi_{i}\left(t\right)+\frac{1}{2}\sum_{i=1}^{N}\xi_{i}^{T}\left(t\right)\left(D_{i}D_{i}{}^{T}+\left(l^{2}+2\right)I_{n}-2r_{i}I_{n}\right)\xi_{i}\left(t\right)+c\sum_{i=1}^{N}\sum_{j=1}^{N}G_{ij}\left(t\right)\xi_{i}^{T}\left(t\right)\xi_{j}\left(t\right)-c\sum_{i=1}^{N}\sum_{\left(i,j\right)\in \tilde{E}}^{}\tilde{\tau_{ij}}\xi_{i}^{T}\left(t\right)\xi_{j}\left(t\right)\\ +\omega^{2}+\frac{1}{2}\sum_{i=1}^{N}\sum_{\left(i,j\right)\in \tilde{E}}^{}\left(-2\beta c\right)e^{-2\beta t}\frac{{\left(L_{ij}\left(t\right)+\tilde{L_{ij}}\right)}^{2}}{2\kappa_{ij}}+\frac{1}{2}\sum_{i=1}^{N}\left(-2\beta c\right)e^{-2\beta t}\frac{{\left(r_{i}\left(t\right)-r_{i}\right)}^{2}}{\gamma_{i}} \end{array}$$

$$\begin{array}{l} \dot{V}\left(t\right)\leq \xi^{T}\left(t\right)\left(C+\frac{1}{2}\left(DD^{T}+I_{N}⊗\left(l^{2}+2\right)I_{n}-2\left(R⊗I_{n}\right)\right)+c\left(G⊗I_{n}\right)-c\lambda_{2}\left(\tilde{\Omega }\right)\left(I_{N}⊗I_{n}\right)+\beta \left(I_{N}⊗I_{n}\right)\right)\xi \left(t\right)\\ -\beta \xi^{T}\left(t\right)\xi \left(t\right)+\omega^{2}+\frac{1}{2}\sum_{i=1}^{N}\sum_{\left(i,j\right)\in \tilde{E}}^{}\left(-2\beta c\right)e^{-2\beta t}\frac{{\left(L_{ij}\left(t\right)+\tilde{L_{ij}}\right)}^{2}}{2\kappa_{ij}}+\frac{1}{2}\sum_{i=1}^{N}\left(-2\beta c\right)e^{-2\beta t}\frac{{\left(r_{i}\left(t\right)-r_{i}\right)}^{2}}{\gamma_{i}}\\ \leq -2\beta \left(\frac{1}{2}\sum_{i=1}^{N}\xi_{i}^{T}\left(t\right)\xi_{i}\left(t\right)+\frac{1}{2}\sum_{i=1}^{N}\sum_{\left(i,j\right)\in \tilde{E}}^{}ce^{-2\beta t}\frac{{\left(L_{ij}\left(t\right)+\tilde{L_{ij}}\right)}^{2}}{2\kappa_{ij}}+\frac{1}{2}\sum_{i=1}^{N}ce^{-2\beta t}\frac{{\left(r_{i}\left(t\right)-r_{i}\right)}^{2}}{\gamma_{i}}\right)+\omega^{2}\\ =-g_{1}V\left(t\right)+\omega^{2} \end{array}$$

where $G={\left(G_{ij}\right)}_{N\times N}$.

When nT+σT≤t≤(n+1)T, it is similar to Theorem 1. And the rest of the proof is the same as Theorem 1. □

**Pinning** **3.** *We consider the control protocol (33) and adaptive laws (11) and (36).*

**Theorem** **4.** *If the HMAS satisfies Assumptions 1–4, the HMAS can achieve QC under the adaptive control protocol (33) and adaptive laws (11) and (36)*.

**Proof.**  When ||ξ||>ε, $\pi_{i}=1$, we construct the following Lyapunov function to achieve the QC of leader-following HMASs:

(40) $$V\left(t\right)=\frac{1}{2}\sum_{i=1}^{N}\xi_{i}^{T}\left(t\right)\xi_{i}\left(t\right)+\frac{1}{2}\sum_{i=1}^{N}\sum_{\left(i,j\right)\in \tilde{E}}^{}ce^{-2\beta t}\frac{{\left(L_{ij}\left(t\right)+\tilde{L_{ij}}\right)}^{2}}{2\kappa_{ij}}+\frac{1}{2}\sum_{i=1}^{N_{S}}ce^{-2\beta t}\frac{{\left(r_{i}\left(t\right)-r_{i}\right)}^{2}}{\gamma_{i}}$$

when nT≤t≤nT+σT.

Taking the derivative of (40), we have

$$\begin{array}{l} \dot{V}\left(t\right)=\sum_{i=1}^{N}\xi_{i}^{T}\left(t\right){\dot{\xi }}_{i}\left(t\right)+\frac{1}{2}\sum_{i=1}^{N}\sum_{\left(i,j\right)\in \tilde{E}}^{}\left(-2\beta c\right)e^{-2\beta t}\frac{{\left(L_{ij}\left(t\right)+\tilde{L_{ij}}\right)}^{2}}{2\kappa_{ij}}+\sum_{i=1}^{N}\sum_{\left(i,j\right)\in \tilde{E}}^{}ce^{-2\beta t}\frac{\left(L_{ij}\left(t\right)+\tilde{L_{ij}}\right)}{2\kappa_{ij}}\dot{L_{ij}}\left(t\right)\\ +\frac{1}{2}\sum_{i=1}^{N_{S}}\left(-2\beta c\right)e^{-2\beta t}\frac{{\left(r_{i}\left(t\right)-r_{i}\right)}^{2}}{\gamma_{i}}+\sum_{i=1}^{N_{S}}ce^{-2\beta t}\frac{\left(r_{i}\left(t\right)-r_{i}\right)}{\gamma_{i}}\dot{r_{i}}\left(t\right) \end{array}$$

$$\begin{array}{l} \dot{V}\left(t\right)\leq \sum_{i=1}^{N}\xi_{i}^{T}\left(t\right)C_{i}\xi_{i}\left(t\right)+\frac{1}{2}\sum_{i=1}^{N}\xi_{i}^{T}\left(t\right)\left(D_{i}D_{i}{}^{T}+\left(l^{2}+2\right)I_{n}-2\partial_{i}r_{i}I_{n}\right)\xi_{i}\left(t\right)+c\sum_{i=1}^{N}\sum_{j=1}^{N}G_{ij}\left(t\right)\xi_{i}^{T}\left(t\right)\xi_{j}\left(t\right)-c\sum_{i=1}^{N}\sum_{\left(i,j\right)\in \tilde{E}}^{}\tilde{\tau_{ij}}\xi_{i}^{T}\left(t\right)\xi_{j}\left(t\right)\\ +\omega^{2}+\frac{1}{2}\sum_{i=1}^{N}\sum_{\left(i,j\right)\in \tilde{E}}^{}\left(-2\beta c\right)e^{-2\beta t}\frac{{\left(L_{ij}\left(t\right)+\tilde{L_{ij}}\right)}^{2}}{2\kappa_{ij}}+\frac{1}{2}\sum_{i=1}^{N_{S}}\left(-2\beta c\right)e^{-2\beta t}\frac{{\left(r_{i}\left(t\right)-r_{i}\right)}^{2}}{\gamma_{i}} \end{array}$$

$$\begin{array}{l} \dot{V}\left(t\right)\leq \xi^{T}\left(t\right)\left(C+\frac{1}{2}\left(DD^{T}+I_{N}⊗\left(l^{2}+2\right)I_{n}-2\left(\tilde{R}⊗I_{n}\right)\right)+c\left(G⊗I_{n}\right)-c\lambda_{2}\left(\tilde{\Omega }\right)\left(I_{N}⊗I_{n}\right)+\beta \left(I_{N}⊗I_{n}\right)\right)\xi \left(t\right)\\ -\beta \xi^{T}\left(t\right)\xi \left(t\right)+\omega^{2}+\frac{1}{2}\sum_{i=1}^{N}\sum_{\left(i,j\right)\in \tilde{E}}^{}\left(-2\beta c\right)e^{-2\beta t}\frac{{\left(L_{ij}\left(t\right)+\tilde{L_{ij}}\right)}^{2}}{2\kappa_{ij}}+\frac{1}{2}\sum_{i=1}^{N_{S}}\left(-2\beta c\right)e^{-2\beta t}\frac{{\left(r_{i}\left(t\right)-r_{i}\right)}^{2}}{\gamma_{i}}\\ \leq -2\beta \left(\frac{1}{2}\sum_{i=1}^{N}\xi_{i}^{T}\left(t\right)\xi_{i}\left(t\right)+\frac{1}{2}\sum_{i=1}^{N}\sum_{\left(i,j\right)\in \tilde{E}}^{}ce^{-2\beta t}\frac{{\left(L_{ij}\left(t\right)+\tilde{L_{ij}}\right)}^{2}}{2\kappa_{ij}}+\frac{1}{2}\sum_{i=1}^{N_{S}}ce^{-2\beta t}\frac{{\left(r_{i}\left(t\right)-r_{i}\right)}^{2}}{\gamma_{i}}\right)+\omega^{2}\\ =-g_{1}V\left(t\right)+\omega^{2} \end{array}$$

where $G={\left(G_{ij}\right)}_{N\times N}$, $\tilde{R}=diag\left(r_{1},r_{2},\ldots ,r_{N_{S}},0,\ldots ,0\right)$.

When nT+σT≤t≤(n+1)T, it is similar to Theorem 1. And the rest of the proof is the same as Theorem 1. □

## 4. Numerical Examples

In this part, we will prove the effectiveness of the proposed control protocol using several simulation examples. Assume that the HMAS contains one leader agent and five follower agents. For the leader system (7), assume the external disturbance is $\psi \left(t\right)={\left(0,0,0\right)}^{T}$. For the follower system (8), the external disturbance is defined as $\varpi_{i}\left(t\right)={\left(0.1\sin t\cos t,0.2\sin t,0.3\cos t\right)}^{T}$.

**Example** **1.** Assume the agent dynamics are described by a classical Chua circuit system model.

In the leader system (7), the linear part is presented as $z_{0}(t)={\left(z_{01},z_{02},z_{03}\right)}^{T}$, and the nonlinear part is presented as $f(z_{0},t)={\left(0.5\left(\left|z_{01}+1|-|z_{01}-1\right|\right),0,0\right)}^{T}$. The system matrix selection is $C_{0}=\left(\begin{array}{ccc} -2.5 & 10 & 0\\ 1 & -1 & 1\\ 0 & -18 & 0 \end{array}\right)$, $D_{0}=\left(\begin{array}{ccc} \frac{35}{6} & 0 & 0\\ 0 & 0 & 0\\ 0 & 0 & 0 \end{array}\right)$. For the follower system (8), the linear part can be described as $z_{i}(t)={\left(z_{i1},z_{i2},z_{i3}\right)}^{T}$, and the nonlinear part can be described as $f(z_{i},t)={\left(0.5\left(\left|z_{i1}+1|-|z_{i1}-1\right|\right),0,0\right)}^{T}$. Assume that the matrix of the follower system is

$$C_{i}=\left(\begin{array}{ccc} -2.5+0.2i & 10+0.3i & 0\\ 1+0.1i & -1+0.1i & 1+0.1i\\ 0 & -18+0.3i & 0 \end{array}\right),\;D_{i}=\left(\begin{array}{ccc} \frac{35}{6} & 0 & 0\\ 0 & 0 & 0\\ 0 & 0 & 0 \end{array}\right)$$

**Figure 1:**
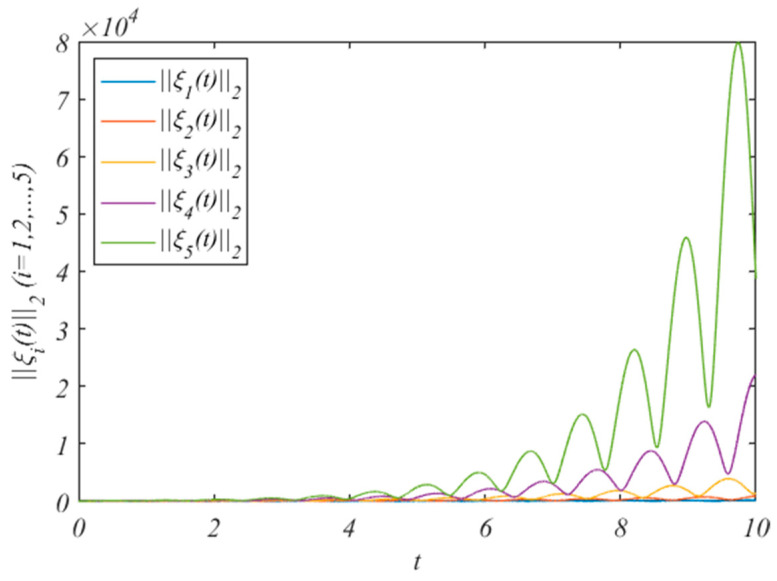
The errors $||\xi_{i}\left(t\right)|{|}_{2}$without adding control protocols.

For state variables, we choose the initial value $z_{0}(0)={\left(2.9,0.75,0.1\right)}^{T}$, and $z_{i}(0)={\left(10+2i,4+i,5+2i\right)}^{T}$. The state changes of the HMASs without the control protocol are shown in Figure 1. It can be concluded from Figure 1 that, when we do not add control protocols, the state changes of the system increase.

**Figure 2:**
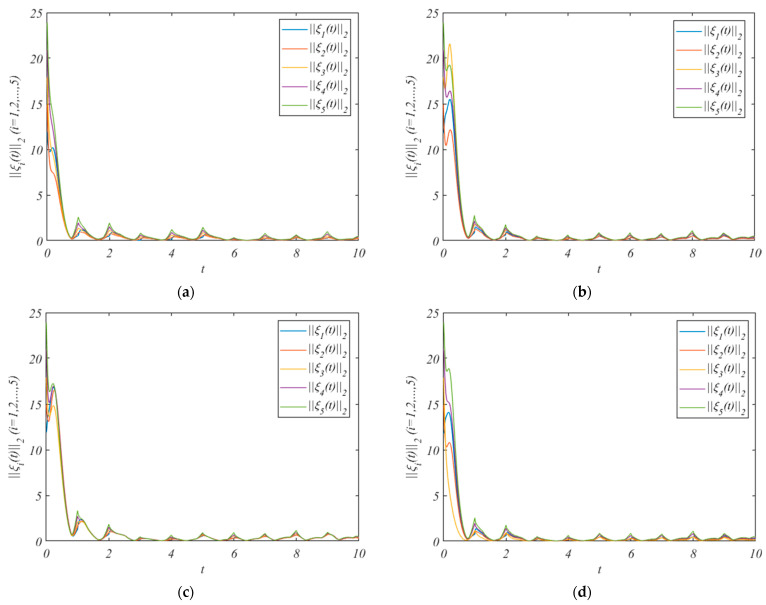
The errors $||\xi_{i}\left(t\right)|{|}_{2}$ under the control protocol. (**a**) is the errors without pinning; (**b**) is the errors under the pinning 1; (**c**) is the errors under the pinning 2; (**d**) is the errors under the pinning 3.

The simulation results after adding the control protocol are shown in Figure 2.

Figure 2a shows the errors under the adaptive control protocol (10) and adaptive laws (11) and (12). We pick the arbitrary value $r(0)={\left(3.3,4.1,2.8,1.6,1.9\right)}^{T}$, and β=0.1$\gamma_{i}=\left(0.010,0.011,0.012,0.013,0.014\right)$, $\kappa_{12}=\kappa_{21}=1.7,\kappa_{13}=\kappa_{31}=1.5$, $\kappa_{14}=\kappa_{41}=1.9$ $\kappa_{15}=\kappa_{51}=1.3,$$\kappa_{24}=\kappa_{42}=1.5,\kappa_{45}=\kappa_{54}=1.5$. It can be concluded from Figure 2a that the errors of the leader system (7) and follower system (8) converge to a bounded range. And the HMAS can achieve QC via the adaptive control protocol (10) and adaptive laws (11) and (12).

Figure 2b shows the errors under the adaptive control protocol (33) and adaptive laws (11) and (12). Assume that the leader only exchanges information with nodes 1 and nodes 2. We pick the arbitrary values $r(0)={\left(2.9,3.6\right)}^{T}$, and β=0.1$\gamma_{i}=\left(0.10,0.11\right)$, $\kappa_{12}=\kappa_{21}=1.7,\kappa_{13}=\kappa_{31}=1.5,\kappa_{14}=\kappa_{41}=1.9$, $\kappa_{15}=\kappa_{51}=1.3,\kappa_{24}=\kappa_{42}=1.5$, and $\kappa_{45}=\kappa_{54}=1.5$.

Figure 2c shows the errors under the adaptive control protocol (10) and adaptive laws (11), (12), and (36). We pick the arbitrary value $r(0)={\left(1.5,1.3,1.6,1.1,1.2\right)}^{T}$, and β=0.1$\gamma_{i}=\left(0.010,0.011,0.012,0.013,0.014\right)$, $\kappa_{14}=\kappa_{41}=1.9$, $\kappa_{15}=\kappa_{51}=1.3,\kappa_{24}=\kappa_{42}=1.5$.

Figure 2d shows the errors under the adaptive control protocol (33) and adaptive laws (11) and (36). Assume that the leader only exchanges information with nodes 1 and nodes 2. We pick the arbitrary value $r(0)={\left(2.7,3.5,3.3\right)}^{T}$, and β=0.1 $\gamma_{i}=\left(0.10,0.11,0.12\right)$, $\kappa_{14}=\kappa_{41}=1.9,$ $\kappa_{15}=\kappa_{51}=1.5$, and $\kappa_{24}=\kappa_{42}=1.5$.

It can be concluded from Figure 2b–d that the errors of the leader system (7) and follower system (8) converge to a bounded range. And the HMASs can achieve QC by using three saturated adaptive pinning control protocols.

**Example** **2.** Assume the agent dynamics are described by a classical Chen circuit system model.

For the leader system (7), the linear part is presented as $z_{0}(t)={\left(z_{01},z_{02},z_{03}\right)}^{T}$ and the nonlinear part is presented as $f(z_{0},t)={\left(0,z_{01}z_{03},z_{01}z_{02}\right)}^{T}$. The system matrix selection is $C_{0}=\left(\begin{array}{ccc} 28 & -28 & 0\\ 7 & -35 & 0\\ 0 & -3 & 0 \end{array}\right)$, $D_{0}=\left(\begin{array}{ccc} 1 & 0 & 0\\ 0 & 1 & 0\\ 0 & 0 & 1 \end{array}\right)$. For the follower system (8), the linear part can be described as $z_{i}(t)={\left(z_{i1},z_{i2},z_{i3}\right)}^{T}$ and the nonlinear part can be described as $f(z_{i},t)={\left(0,z_{i1}z_{i3},z_{i1}z_{i2}\right)}^{T}$. Assume that the matrix of the follower system is

$$C_{i}=\left(\begin{array}{ccc} 28+i & -28+i & 0\\ 7+i & -35+2i & 0\\ 0 & -3+i & 0 \end{array}\right),\;D_{i}=\left(\begin{array}{ccc} 1 & 0 & 0\\ 0 & 1 & 0\\ 0 & 0 & 1 \end{array}\right)$$

**Figure 3:**
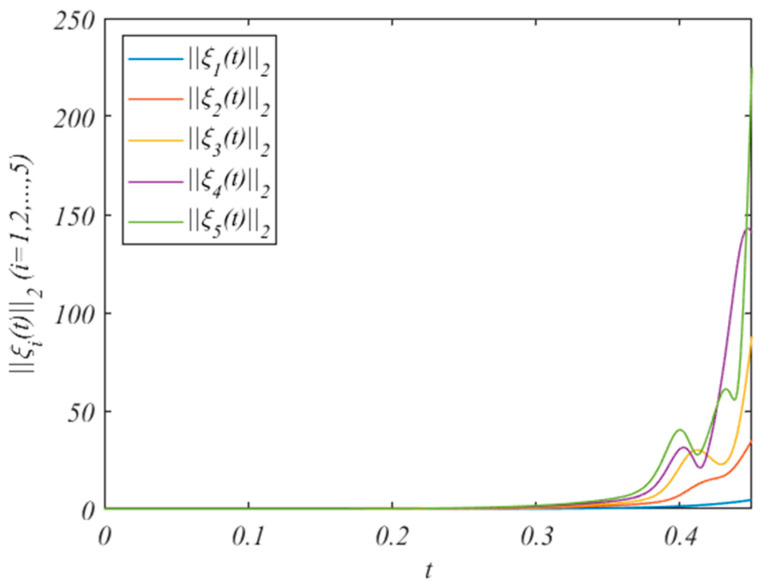
The errors $||\xi_{i}\left(t\right)|{|}_{2}$ without adding control protocols.

For state variables, we choose the initial value $z_{0}(0)={\left(-9,14,20\right)}^{T}$, and $z_{i}(0)={\left(-9+2i,-14+i,20-i\right)}^{T}$. The state changes of the HMAS without the control protocol are shown in Figure 3. It can be concluded from Figure 3 that when we do not add control protocols, the state changes of the system increase.

**Figure 4:**
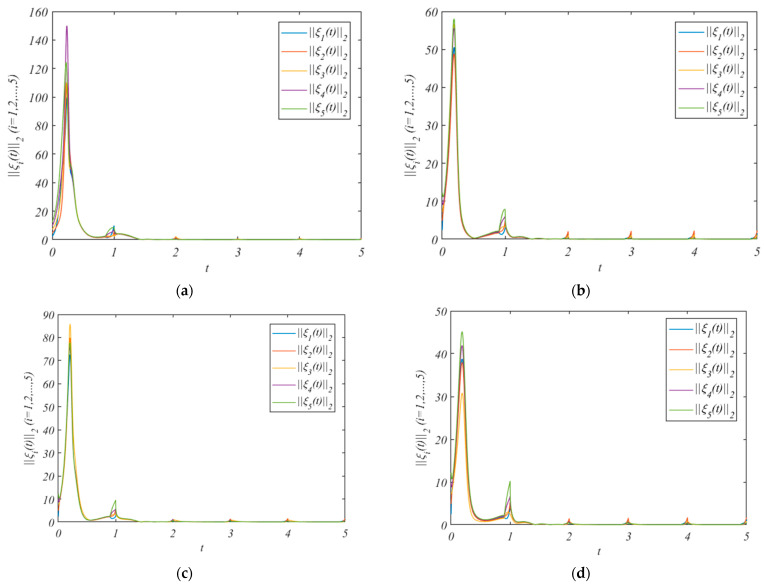
The errors $||\xi_{i}\left(t\right)|{|}_{2}$ under the control protocol. (**a**) is the errors without pinning; (**b**) is the errors under the pinning 1; (**c**) is the errors under the pinning 2; (**d**) is the errors under the pinning 3.

The simulation results after adding the control protocol are shown in Figure 4.

Figure 4a shows the errors under the adaptive control protocol (10) and adaptive laws (11) and (12). We pick the arbitrary value $r(0)={\left(2.7,3.2,2.4,3.1,1.5\right)}^{T}$, and β=0.1$\gamma_{i}=\left(0.010,0.011,0.012,0.013,0.014\right)$, $\kappa_{12}=\kappa_{21}=1.7,\kappa_{13}=\kappa_{31}=1.5$, $\kappa_{14}=\kappa_{41}=1.9$ $\kappa_{15}=\kappa_{51}=1.3,$ $\kappa_{24}=\kappa_{42}=1.5,\kappa_{45}=\kappa_{54}=1.5$. It can be concluded from Figure 4a that the errors of the leader system (7) and follower system (8) converge to a bounded range. And the HMASs can achieve QC by using the adaptive control protocol (10) and adaptive laws (11) and (12).

Figure 4b shows the errors under the adaptive control protocol (33) and adaptive laws (11) and (12). Assume that the leader only exchanges information with nodes 1 and nodes 2. We pick the arbitrary value $r(0)={\left(2.7,3.2\right)}^{T}$, and β=0.1 $\gamma_{i}=\left(0.10,0.11\right)$, $\kappa_{12}=\kappa_{21}=1.7,\kappa_{13}=\kappa_{31}=1.5,\kappa_{14}=\kappa_{41}=1.9$, $\kappa_{15}=\kappa_{51}=1.3,\kappa_{24}=\kappa_{42}=1.5$, and $\kappa_{45}=\kappa_{54}=1.5$.

Figure 4c shows the errors under the adaptive control protocol (10) and adaptive laws (11), (12), and (36). We pick the arbitrary value $r(0)={\left(2.7,3.2,2.4,3.1,1.5\right)}^{T}$, and β=0.1 $\gamma_{i}=\left(0.010,0.011,0.012,0.013,0.014\right)$, $\kappa_{14}=\kappa_{41}=1.9$, $\kappa_{15}=\kappa_{51}=1.3,\kappa_{24}=\kappa_{42}=1.5$.

Figure 4d shows the errors under the adaptive control protocol (33) and adaptive laws (11) and (36). Assume that the leader only exchanges information with nodes 1 and nodes 2. We pick the arbitrary value $r(0)={\left(2.7,3.2,2.4\right)}^{T}$, and β=0.1 $\gamma_{i}=\left(0.10,0.11,0.12\right)$, $\kappa_{14}=\kappa_{41}=1.9,$ $\kappa_{15}=\kappa_{51}=1.5$, and $\kappa_{24}=\kappa_{42}=1.5$.

It can be concluded from Figure 4b–d that the errors of the leader system (7) and follower system (8) converge to a bounded range. And the HMASs can achieve QC by using the three saturated adaptive pinning control protocols.

**Example** **3.** Assume the agent dynamics are described by a manipulator system with flexible joints model, whose dynamic system is as follows:

(41) $$\{\begin{array}{c} \dot{\theta_{m}}=\omega_{m}\\ \dot{\omega_{m}}=\frac{k}{J_{m}}\left(\theta_{1}-\theta_{m}\right)-\frac{B}{J_{m}}\omega_{m}+\frac{k_{\tau }}{J_{m}}u\\ \dot{\theta_{1}}=\omega_{1}\\ \dot{\omega_{1}}=-\frac{k}{J_{1}}\left(\theta -\theta_{m}\right)-\frac{mgh}{J_{1}}\sin\left(\theta_{1}\right) \end{array}$$

Let $z={\left(\begin{array}{cccc} \theta_{m} & \omega_{m} & \theta_{1} & \omega_{1} \end{array}\right)}^{T}$. The nonlinear dynamics of the robotic arm system are equivalent to

(42) $$\dot{z}\left(t\right)=Az\left(t\right)+f\left(z\right)+g\left(y\right)u\left(t\right)$$

Let $g\left(y\right)=I_{n}$. For the leader system (7), the linear part is presented as $z_{0}(t)={\left(z_{01},z_{02},z_{03},z_{04}\right)}^{T}$ and the nonlinear part is presented as $f(z_{0},t)=(0,0,0,\frac{1}{3}\sin{\left(z_{03}{}^{3}\right)}^{T}$. The system matrix selection is $C_{0}=\left(\begin{array}{cccc} 0 & 1 & 0 & 0\\ -48.6 & -1.25 & 48.6 & 0\\ 0 & 0 & 0 & 1\\ 19.5 & 0 & -19.5 & 0 \end{array}\right)$, $D_{0}=\left(\begin{array}{cccc} 1 & 0 & 0 & 0\\ 0 & 1 & 0 & 0\\ 0 & 0 & 1 & 0\\ 0 & 0 & 0 & 1 \end{array}\right)$. For the follower system (8), the linear part can be described as $z_{i}(t)={\left(z_{i1},z_{i2},z_{i3}\right)}^{T}$ and the nonlinear part can be described as $f(z_{i},t)=(0,0,0,\frac{1}{3}\sin{\left(z_{i3}{}^{3}\right)}^{T}$. Assume that the matrix of the follower system is

$$C_{i}=\left(\begin{array}{cccc} 0 & 1+0.1i & 0 & 0\\ -48.6+0.5i & -1.25+0.1i & 48.6+0.6i & 0\\ 0 & 0 & 0 & 1+0.2i\\ 19.5+0.3i & 0 & -19.5+0.2i & 0 \end{array}\right),\;D_{i}=\left(\begin{array}{cccc} 1 & 0 & 0 & 0\\ 0 & 1 & 0 & 0\\ 0 & 0 & 1 & 0\\ 0 & 0 & 0 & 1 \end{array}\right)$$

**Figure 5:**
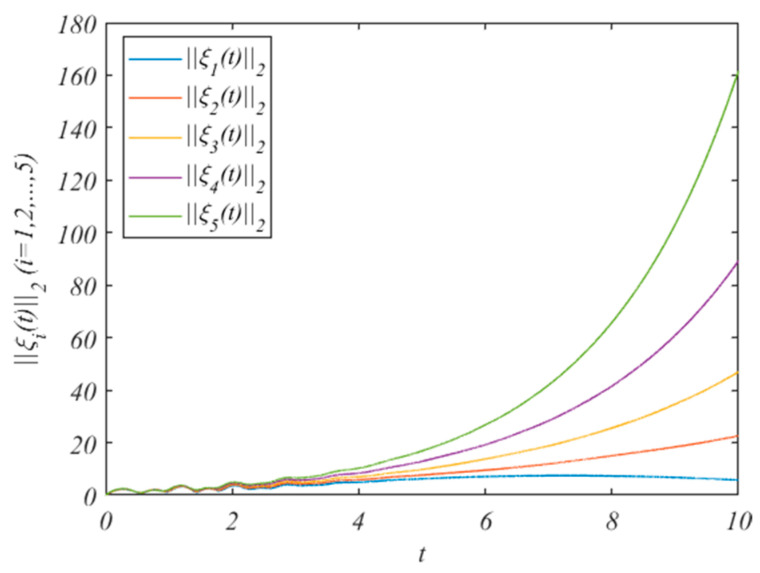
The errors $||\xi_{i}\left(t\right)|{|}_{2}$ without adding control protocols.

For state variables, we choose the initial value $z_{0}(0)={\left(0.2,0.5,0.7,0.3\right)}^{T}$, and $z_{i}(0)={\left(0.3+1.2i,0.5+1.1i,0.8+1.3i,0.4+1.5i\right)}^{T}$. The state changes of the HMASs without the control protocol are shown in Figure 5. It can be concluded from Figure 5 that, when we do not add control protocols, the state changes of the system increase.

**Figure 6:**
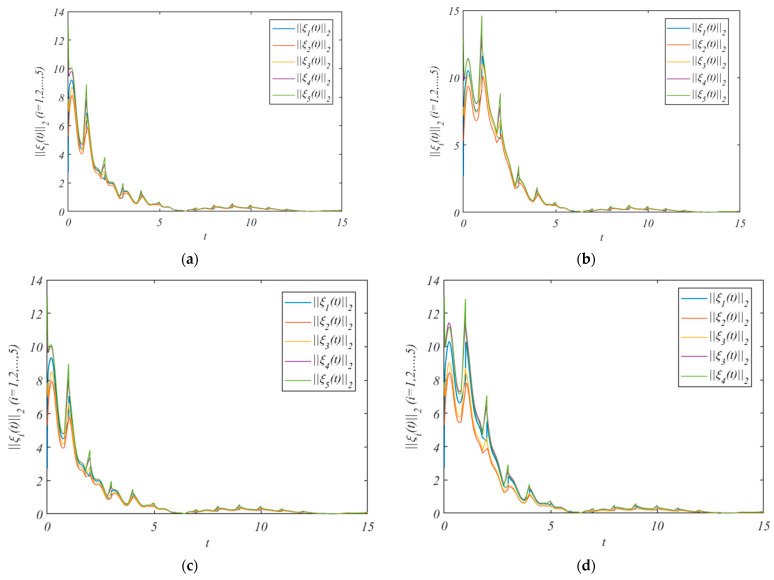
The errors $||\xi_{i}\left(t\right)|{|}_{2}$ under the control protocol. (**a**) is the errors without pinning; (**b**) is the errors under the pinning 1; (**c**) is the errors under the pinning 2; (**d**) is the errors under the pinning 3.

The simulation results after adding the control protocol are shown in Figure 6.

Figure 6a shows the errors under the adaptive control protocol (10) and adaptive laws (11) and (12). We pick the arbitrary value $r(0)={\left(1.3,2.1,0.4,0.7,0.1\right)}^{T}$, and β=0.1 $\gamma_{i}=\left(0.010,0.009,0.010,0.008,0.012\right)$, $\kappa_{12}=\kappa_{21}=0.3,\kappa_{13}=\kappa_{31}=0.3$, $\kappa_{14}=\kappa_{41}=0.45$ $\kappa_{15}=\kappa_{51}=0.55,$ $\kappa_{23}=\kappa_{32}=0.65,\kappa_{25}=\kappa_{52}=0.6,\kappa_{34}=\kappa_{43}=0.4$. It can be concluded from Figure 6a that the errors of the leader system (7) and follower system (8) converge to a bounded range. And the HMASs can achieve QC when using the adaptive control protocol (10) and adaptive laws (11) and (12).

Figure 6b shows the errors under the adaptive control protocol (33) and adaptive laws (11) and (12). Assume that the leader only exchanges information with nodes 1 and nodes 2. We pick the arbitrary value $r(0)={\left(1.3,2.1\right)}^{T}$, and β=0.1 $\gamma_{i}=\left(0.010,0.009\right)$, $\kappa_{12}=\kappa_{21}=0.3,\kappa_{13}=\kappa_{31}=0.3$, $\kappa_{14}=\kappa_{41}=0.45$, and $\kappa_{15}=\kappa_{51}=0.55$.

Figure 6c shows the errors under the adaptive control protocol (10) and adaptive laws (11), (12), and (36). We pick the arbitrary value $r(0)={\left(1.3,2.1,0.4,0.7,0.1\right)}^{T}$, and β=0.1 $\gamma_{i}=\left(0.010,0.009,0.010,0.008,0.012\right)$, $\kappa_{14}=\kappa_{41}=1.9$, $\kappa_{15}=\kappa_{51}=1.3,\kappa_{24}=\kappa_{42}=1.5$.

Figure 6d shows the errors under the adaptive control protocol (33) and adaptive laws (11) and (36). Assume that the leader only exchanges information with nodes 1 and nodes 2. We pick the arbitrary value $r(0)={\left(2.7,3.5,3.3\right)}^{T}$, and β=0.1$\gamma_{i}=\left(0.010,0.009,0.010\right)$, $\kappa_{14}=\kappa_{41}=1.9,$ $\kappa_{15}=\kappa_{51}=1.3$, and $\kappa_{24}=\kappa_{42}=1.5$.

It can be concluded from Figure 6b–d that the errors of the leader system (7) and follower system (8) converge to a bounded range. And the HMASs can achieve QC by using three saturated adaptive pinning control protocols.

From the above three simulations, we can see the effectiveness of our proposed saturated adaptive control protocol, and it can be widely used in various models. From the simulations of three saturated adaptive pinning control protocols, it can be clearly seen that distributed control has high robustness. By appropriately increasing the coupling strength or feedback gain, the error caused by the loss of control can be compensated for.

**Remark** **2.** *Like existing works* [11,12,13,14,15,16,17,18,19,20,21,22,23,24,25,26,27,28,29,30,31]*, we deal with this open problem with theoretical derivation and numerical simulation. It is worth noting that, to expand the application scope of the proposed control method, we chose a universal unified multi-agent system model, without establishing a system model for specific applications. At the same time, to reduce the complexity of the control method and the theoretical derivation, we simplified the system appropriately and adopted a more idealized normalized model. From the perspective of control method research, it is reasonable and feasible to verify the effectiveness and correctness of the proposed control methods via numerical simulation. Within our research framework, one can construct a system model for a specific application by considering suitable environment and detailed parameters for specific applications, such as multi-robot formation and multi-UAV formation. In addition, to facilitate engineering applications, we should consider more complex factors and more realistic working conditions, such as limited communication, unpredictable state, unknown parameters, perturbations, time delays, and so on. In this way, the proposed control methods might be practically tested and verified through real-world experiments.*

## 5. Conclusions

The QC of nonlinear HMASs with an external interference is studied in this paper. Firstly, we design a periodic intermittent adaptive control protocol with saturation, which controls both the internal coupling between follower agents and the communication between the leader and follower. Then, the feasibility of the control protocol is proved theoretically using the Lyapunov function method. The convergence range of the QC error is obtained by using lemma and inequality techniques. Furthermore, three cost-saving saturated adaptive pinning control protocols are proposed. Adaptive feedback gain is applied to only some of the followers, and only some the followers can interact with each other. Due to the coupling effect inside the HMAS, the QC of the whole HMAS can be achieved as long as each agent is connected. The adaptive check control protocol greatly saves on control costs and demonstrates the high robustness of the distributed control. Finally, the correctness of the control protocol is proved through four numerical simulations. Although this paper presents a novel and reasonable control method, it is still difficult to fully apply it in practical engineering applications. The main reason for this is that the model is not fully consistent with the actual model. Therefore, in future work, in order to make the research method and results more practical, we will apply the theoretical method to the collaborative control of UAVs and robots as the focus of our next study.

## Data Availability

Not applicable.
